# Widespread clinical implementation of the teen online problem-solving program: Progress, barriers, and lessons learned

**DOI:** 10.3389/fresc.2022.1089079

**Published:** 2023-02-07

**Authors:** Shari L. Wade, Kathleen Walsh, Beth S. Slomine, Kimberly C. Davis, Cherish Heard, Brianna Maggard, Melissa Sutcliffe, Marie Van Tubbergen, Kelly McNally, Kathleen Deidrick, Michael W. Kirkwood, Ann Lantagne, Sharon Ashman, Shannon Scratch, Gayle Chesley, Bethany Johnson-Kerner, Abigail Johnson, Lindsay Cirincione, Cynthia Austin

**Affiliations:** ^1^ Department of Pediatrics, Cincinnati Children's Hospital Medical Center, University of Cincinnati, Cincinnati, OH, United States; ^2^ Department of General Pediatrics, Boston Children's Hospital, Harvard University, Boston, MA, United States; ^3^ Department of Psychiatry and Behavioral Sciences, Kennedy Krieger Institute, Johns Hopkins University School of Medicine, Baltimore, MD, United States; ^4^ Department of Pediatrics, Texas Children's Hospital, Baylor College of Medicine, Houston, TX, United States; ^5^ Department of Psychology University of Cincinnati Cincinnati, OH, United States; ^6^ Department of Pediatrics, Cincinnati Children's Hospital Medical Center, Cincinnati, OH, United States; ^7^ Department of Physical Medicine and Rehabilitation, University of Pittsburgh, Pittsburgh, PA, United States; ^8^ Department of Physical Medicine and Rehabilitation, Michigan Medicine, University of Michigan, Ann Arbor, MI, United States; ^9^ Department of Pediatrics, Nationwide Children's Hospital, The Ohio State University, Columbus, OH, United States; ^10^ Neurology and Behavioral Psychology, Pediatrics Neurophysiology, St. Luke's Children's Hospital, Boise, ID, United States; ^11^ Department of Rehabilitation Medicine, Children's Hospital Colorado, University of Colorado School of Medicine, Aurora, CO, United States; ^12^ Department of Rehabilitation Medicine, Seattle Children's Hospital, University of Washington, Seattle, WA, United States; ^13^ Bloorview Research Institute, Holland Bloorview Kids Rehabilitation Hospital, Toronto, ON, Canada; ^14^ The Department of Child & Adolescent Psychiatry and Behavioral Sciences, Children's Hospital of Philadelphia, Perelman School of Medicine at the University of Pennsylvania, Philadelphia, PA, United States; ^15^ Pediatric Neurology, Benioff Children's Hospital, University of California at San Francisco, San Francisco, CA, United States; ^16^ Department of Neurology, Dell Children's Medical Center, University of Texas at Austin Dell Medical School, Austin, TX, United States

**Keywords:** traumatic brain injury, pediatric injury, implementation, telehealth, problem solving, acquired brain injury

## Abstract

**Objective:**

We describe the clinical implementation in North America of Teen Online Problem Solving (TOPS), a 10+ session, evidence-based telehealth intervention providing training in problem-solving, emotion regulation, and communication skills.

**Methods:**

Twelve children's hospitals and three rehabilitation hospitals participated, agreeing to train a minimum of five therapists to deliver the program and to enroll two patients with traumatic brain injuries (TBI) per month. Barriers to reach and adoption were addressed during monthly calls, resulting in expansion of the program to other neurological conditions and extending training to speech therapists.

**Results:**

Over 26 months, 381 patients were enrolled (199 TBI, 182 other brain conditions), and 101 completed the program. A total of 307 therapists were trained, and 58 went on to deliver the program. Institutional, provider, and patient barriers and strategies to address them are discussed.

**Conclusions:**

The TOPS implementation process highlights the challenges of implementing complex pediatric neurorehabilitation programs while underscoring potential avenues for improving reach and adoption.

## 1. Introduction

The United States and other countries invest considerable resources annually developing an evidence base to improve the quality and consistency of health care. However, a gap exists between the identification of promising evidence-based practices (EBP) validated through randomized controlled clinical trials and their introduction into clinical care. Evidence suggests that the vast majority of EBPs never move beyond peer-reviewed publications into everyday clinical practice (1). This so-called “valley of death” has generated increasing interest in implementation science and support for efforts to systematically move EBPs into standard clinical care (2).

Pediatric neurorehabilitation suffers from both a dearth of EBPs and unique challenges around efforts to clinically implement them. Most children with behavioral health care needs following traumatic (TBI) or other acquired brain injuries (ABI), such as stroke or brain tumors, fail to receive behavioral health treatments of any type (3, 4). Factors contributing to this gap include difficulty identifying the role of the TBI or ABI in problems, a paucity of providers who have an appreciation of the complex interplay of brain-based impairments and everyday functioning (5, 6) a lack of liaising between medical specialists and community-based personnel (7, 8), and limited access to medical and mental health providers due to family resources issues such as transportation and insurance coverage. Moreover, adolescents and parents may not want behavioral health care services due to perceived stigma around “mental health” and alternative, non-evidence based “medical” approaches, such as brain-training games (9, 10).

Recent evidence-based guidelines for pediatric ABI (10) have identified Teen Online Problem Solving [TOPS], along with Online Family Problem-Solving Treatment [F-PST] and Counselor Assisted Problem Solving [CAPS], developed by Wade and colleagues and hereafter collectively referred to as TOPS, as a practice standard for treating behavior problems, executive function deficits, and family difficulties for adolescents with brain injuries. More than 20 years of research including five randomized controlled trials, a comparative effectiveness trial, and an individual participant meta-analysis provide substantial evidence of the program's efficacy. Outcomes have included improvements in child behavior, executive functioning, and participation in school, home, and community settings, as well as reduced parent-teen conflict and parent depression and distress (11–13). Much of the support for the program's efficacy is based on studies of self-guided completion of online learning modules coupled with videoconference sessions with a therapist. A comparative effectiveness study investigating this delivery model against face-to-face delivery with no website and self-guided delivery through the website alone, however, suggest that all three modes of delivery could be effective in improving executive dysfunction and quality of life (14, 15).

In 2019, an implementation award from the Patient Centered Outcomes Research Institute (PCORI) sought to examine the clinical implementation of the TOPS program content in these three modalities, considering patient choice, insurance coverage for behavioral health care, and the availability of therapists and broad-band internet access. The RE-AIM (Reach Effectiveness Adoption Implementation Maintenance) framework was utilized to examine the numerous facets of implementation. Specifically for the purposes of this investigation, the focus was on characterizing *reach,* defined as the number of patients screened, referred, and treated, and *adoption*, defined as the number of therapists trained and delivering the program (16). Our Reach target was to enroll two patients per site with TBI each month. This goal was extended to four patients per month (two with TBI and two with other conditions) when we expanded to non-TBI diagnoses. For Adoption, our target was to train five therapists per site to deliver the program. Ten sites, including eight children's hospitals and two pediatric rehabilitation hospitals, began implementation in February 2020 and an additional five sites were added in fall 2020.

Our objectives here are to: (1) describe the process of implementation over the initial 26 months including *reach* and *adoption* data, considering the unique context of COVID-19; (2) characterize barriers to reach and adoption, as well as strategies for overcoming those barriers; and (3) report changes in reach and adoption over time. In doing so, we hope to promote and inform further efforts to implement EBPs in pediatric rehabilitation.

## 2. Materials and methods

### 2.1. Program description and development

The TOPS program includes ten core sessions addressing cognitive reframing, metacognitive strategies, self-regulation, and communication skills in a collaborative problem-solving framework. Adolescents and caregivers were encouraged to complete the program together. However, therapists had the option of working with adolescents individually, depending on family circumstances and parental availability (17, 18). For adolescents with greater impairment, emphasis is placed on helping parents understand how they could better support the child through environmental modifications. Supplemental session content focused on concerns faced by some, but not all, adolescents. See prior publications for a more detailed description of the program and session content (17). The TOPS website included learning modules covering the core and supplemental session content along with a problem-solving component that supported families in working through the five-step problem-solving process to create a step-by-step plan for achieving their aim.

In 2019, ten sites, including eight large children's hospitals and two rehabilitation hospitals were identified as initial implementation sites based on their patient volume and interest in providing active neurorehabilitation to their patients. Investigators at these sites actively solicited therapists at their sites who were interested in receiving training and delivering the program. In February 2020, therapists across sites began training, and in May 2020 began using the TOPS program with patients and families (details about the training process are provided separately below).

To expand reach, we created a parallel transdiagnostic version of the website that could be used with other acquired brain injuries (stroke, pediatric brain tumor, infectious and anoxic insults) and brain conditions (e.g., epilepsy) that were associated with substantial executive dysfunction). We used a multistep interactive process for the transdiagnostic website. In August of 2020, therapists began using the existing TOPS program for TBI with adolescents with diagnoses other than TBI and provided feedback regarding the appropriateness of various content and needed modifications. Between July 2020 and January 2021, we conducted key informant interviews with patients and families with a range of neurological conditions (e.g., brain tumors, epilepsy, stroke) who suggested changes to the website. Between January 2021 and March 2021, we also conducted a survey of medical providers regarding the frequency of neurocognitive and socioemotional concerns across diagnoses. This 31 item survey contained 7 items pertaining to physical symptoms (e.g., fatigue, pain); 7 items pertaining to cognitive-communication symptoms (e.g., concentration/attention, processing speed); 8 items pertaining to behavioral or emotional symptoms (e.g., impulsivity, depression); and 7 items pertaining to social and family functioning (e.g., social rejection, family impact/functioning). Ninety-seven providers began the survey and 34 completed it. Responses indicated substantial overlap in concerns across conditions, including high levels of attention problems, executive dysfunction, and school difficulties, with some differences in the profiles of most problematic concerns for each diagnosis (e.g., greater behavioral dysregulation among those with anoxic injuries).

Together, feedback from therapists delivering the existing program, data from the key informant interviews, and provider surveys informed modifications to the website and program delivery. Improvements to the transdiagnostic website included referring to “brain conditions” rather than “brain injuries”, removing references to “before your injury”, incorporating videos of adolescents and young adults with non-TBI neurological diagnoses, adding an alternate core session on managing fears and worries to address anxiety (as an alternative to controlling your anger), and creating additional supplemental modules. New supplemental content focused on making choices, self-advocacy, initiation, managing medications, and coping with visible and invisible disabilities.

Clinicians also proposed modifications to patient-eligibility criteria to further support successful program use in their setting (19). Thus, although the program was validated with adolescents with TBI between 13 and 18 years of age, we determined that clinicians could enroll both younger (i.e. 12), and older (i.e. 20–22), patients who they thought would benefit. Clinicians could also offer the program to adolescents with intellectual disabilities and comorbid neurological diagnoses if they viewed the family as capable of scaffolding the teen's involvement.

### 2.2. Therapist training

Therapists were trained during a 7-hour training delivered either in a single, day-long session or two half days. Training was supported by a detailed therapist's manual and therapist's training website. We sought to train a minimum of five therapists at each site to deliver the program. Initial therapists included pediatric psychologists, neuropsychologists, and psychology interns and fellows. Three therapist trainings were held in person and the remaining trainings were conducted virtually due to the pandemic. Training at all ten original implementation sites was completed in spring 2020. Five additional sites were added and trained in fall 2020, with 15 sites enrolling patients beginning in 2021.

To further expand reach, we developed a multiprong strategy for increasing therapist capacity that was tailored to the site. At sites with active psychology or neuropsychology training programs, we developed processes for integrating TOPS into existing clinical rotations for predoctoral interns and postdoctoral fellows, or creating a distinct TOPS rotation. When successful, this process has provided a pipeline of available therapists for TOPS referrals and allowed closer integration of neuropsychological assessment and treatment.

Secondly, we began training speech language pathologists (SLPs) to deliver the program in the Fall of 2020. As described elsewhere (Lundine, Chitwood, & Wade, under review), prior to training SLPs, the PI worked with a team of SLPs to revise the manual and training materials to reframe emotion regulation issues in the context of cognitive communication challenges and develop resources for knowing when substantial mental health or family concerns might necessitate consultation or referral to a mental health provider.

Finally, in areas with few psychologists/neuropsychologists, training was extended to master's level clinicians in social work and other disciplines. Although this approach expanded therapist availability at these sites, it raised additional challenges about finding time in their schedules to receive consultation/supervision around the neurological underpinnings of the child's behavioral health concerns and how to tailor treatment accordingly.

### 2.3. Plan and metrics

As noted above, we initiated implementation at eight tertiary care children's hospitals and two rehabilitation hospitals. Additional funding from PCORI supported expansion to four additional children's hospitals and one additional pediatric rehabilitation hospital in fall 2020. Initial meetings with each site mapped processes for identifying, screening, and referring patients to the program. We conducted monthly cross-site meetings to discuss barriers and highlight successful implementation strategies beginning in March 2020. These monthly “All Teach All Learn” meetings (20), with the goal of fostering a network of shared knowledge (e.g., templates for chart notes, tip sheets for families for using the website, use of QR codes to link families to the website login), included Site PIs and research support staff (where applicable).

Given findings of comparable efficacy among face-to-face, therapist-guided online, and self-guided only versions of the program (14, 15), implementation sites were encouraged to consider both clinician recommendations and patient and family choice regarding the mode of treatment delivery (15). At the inception of the implementation program, 8 of 10 sites were not offering telehealth services, and consequently patients at those sites could only opt for in-person treatment or self-guided completion of the program online. With the onset of the global pandemic in winter 2020, telehealth practices rapidly evolved (21); by summer 2020, all participating sites were offering telehealth visits. The reintroduction of in-person visits for behavioral health care varied by site and region; however, all sites were again offering in-person visits by spring 2021.

Implementation teams also used the monthly “All Teach All Learn” calls and monthly active therapist calls to identify barriers to implementation and engage in problem solving to develop and review strategies for overcoming barriers. Project PIs also maintained quarterly one-on-one calls with each site beginning in fall 2020 as a strategy to better understand and address site-specific barriers. We also conducted qualitative interviews with a total of three patients and three therapists regarding their experiences completing and delivering the program and barriers that they encountered to further inform our approach to improving patient engagement. Open-ended questions probed difficulties with accessing the website/logging on, understanding and engaging with the online learning module content, and suggestions for improving the program. These data were compiled and analyzed thematically. Strategies to address barriers were identified in a similar fashion. Details, the range of strategies that were identified, and trials are described below. Given the short implementation timeline and considerable heterogeneity across sites and concomitant barriers, we diverged in some respects from the traditional plan-do-study-act cycles associated with quality improvement activities. Specifically, we trialed multiple interventions to improve uptake simultaneously. Additionally, the team at each site determined which strategies to deploy, since not all actions were possible at every site.

To assess initial implementation success, we focused on Reach and Adoption. Reach was defined by number of eligible patients referred and enrolled in the program (target four patients enrolled per site per month). Adoption was defined as the number of therapists trained and the number who went on to deliver the program (target five therapists per site trained to deliver the program). We report data regarding program enrollment (TBI or transdiagnostic), treatment condition (self-guided vs. therapist-supported), and therapist metrics (number trained, number actively treating, trainee involvement).

## 3. Results

### 3.1. Implementation reach and adoption

**Figure 1:**
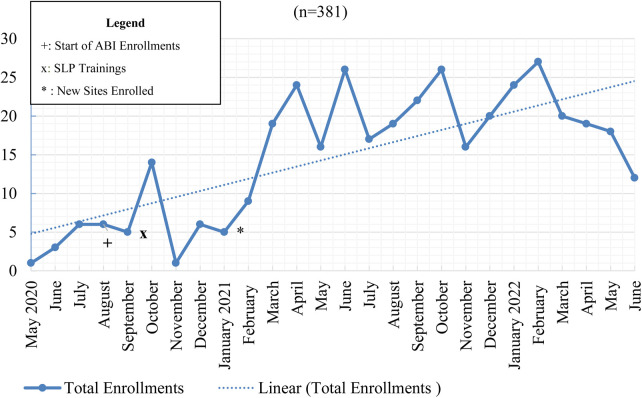
TOPS program enrollments from May 2020 through June 2022.

Figure 1 depicts program enrollment from May 2020, when enrollment began, until June 2022. In September 2020, we began enrolling patients with diagnoses other than TBI and in January 2021, we began enrolling at the five additional sites.

### 3.2. Patient enrollment

We projected enrolling two patients per site per month with TBI, and two patients per site per month with other diagnoses, following expansion to other diagnoses in Fall 2020. New sites joined the TOPS program in January of 2021 and began enrolling patients. As depicted in Figure 1, a total of 381 patients were enrolled in the program over 26 months. Enrollment rates varied substantially over time and across sites, with the total enrolled at the original sites ranging from 9 to 63 and numbers for the additional sites ranging from 9 to 29. Among patients enrolled, 199 (52.23%) had sustained a TBI and 182 (47.77%) had other ABIs or neurological diagnoses including brain tumors, stroke, spina bifida, epilepsy, and brain infection/encephalitis.

### 3.3. Program adherence and completion

Adherence varied between the self-guided and therapist-guided programs. Of the 205 families enrolled in the self-guided treatment through June 2022, 10 (∼5%) completed the program by finishing at least five sessions. 101 (∼49%) completed no sessions over a six-month period. The remainder (*n* = 94; 46%) were actively enrolled. Of the 176 families enrolled in the therapist-guided treatment through June 2022, 94 (∼53%) completed the program by finishing at least five sessions. 52 (29.5%) ended treatment before completing five sessions, and 30 (17%) were still actively involved in the treatment.

### 3.4. Therapist adoption

**Figure 2:**
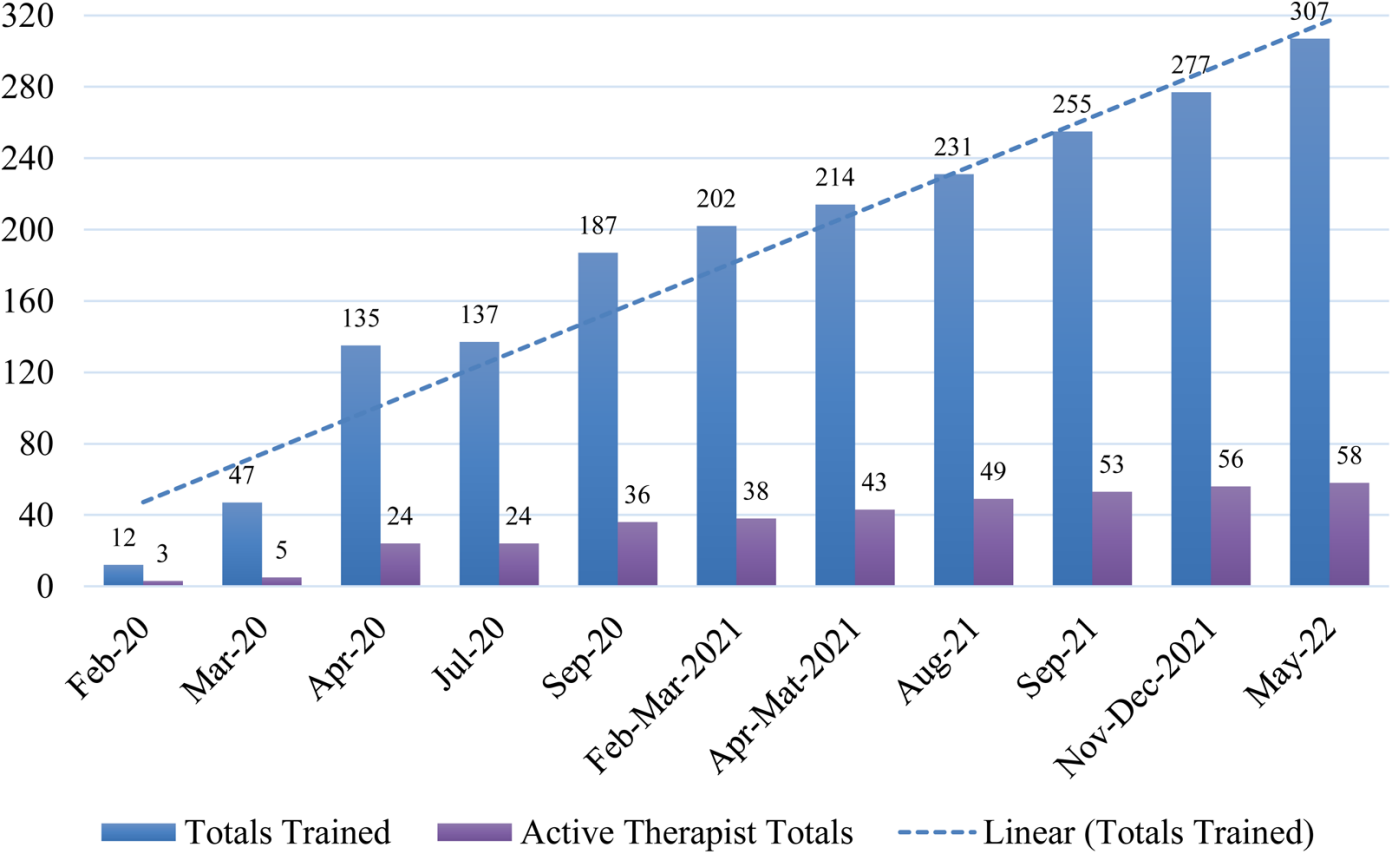
TOPS therapists trained and delivering the program from spring 2020 through spring 2022.

We planned to train a minimum of five therapists per site across 15 sites (75 total) to deliver the program. From February 2020 to spring 2022, 307 providers were trained to deliver the TOPS program at implementation sites. Provider backgrounds included clinical psychologists, neuropsychologists, and varying levels of psychology and neuropsychology trainees. After extending training to SLPs who worked with this population, 38 SLPs completed training through Spring 2022. Figure 2 depicts the number of therapists and SLPs trained and delivering the program over time.

### 3.5. Therapists delivering TOPS

Of the 307 individuals trained, 66 therapists, including 25 clinical psychologists, 14 neuropsychologists, 13 SLPs, 3 social workers, 2 master's level counselors, and 9 psychology trainees, delivered the program to patients. Reasons for not delivering the program after completing training included: (1) completed the training for other purposes (i.e., trained as support team staff, to provide supervision, for educational purposes, to provide appropriate referrals), (2) moved to a different role or position before having an opportunity to deliver the program, and (3) plan to deliver the program but have not yet had the opportunity. Of note, therapists at non-supported sites often experienced considerable lags gaining institutional approval and support that would allow them to deliver the program.

### 3.6. Barriers and strategies

#### 3.6.1. Institutional barriers

Quarterly meetings with implementation teams at each site identified a range of potential barriers including: (1) lack of a medical home or established follow-up clinics for many of the patients with conditions of interest, (2) lack of behavioral health care providers for these populations, (3) lack of communication among subspecialties treating these children about neurobehavioral concerns and associated failure to generate referrals, and (4) lack of broader institutional support/reliance on a single champion. Fragmented follow-up care made patient identification challenging, and this was especially true for rural patients who may be less likely to receive follow-up care at all. Additional potential barriers arose from the age range and severity of patients treated; this was particularly evident at the participating rehabilitation hospitals where substantial numbers of patients were extremely low functioning and unable to engage with the program. Catchment areas that crossed state boundaries posed additional barriers to offering the therapist-guided option of the program, because therapists were most often not licensed in multiple states.

Developing a workflow to integrate TOPS into existing clinical care and getting buy-in from administrative staff to schedule appointments that were seen as different and required different steps (e.g., setting up screen sharing and using a different visit type) from typical psychotherapy appointments posed challenges at some sites. At some institutions, the videoconferencing platform that they used for telehealth did not support screen sharing, requiring additional problem-solving around program delivery. Staff/administrative infrastructure to determine insurance coverage, schedule appointments, and process billing was necessary to support families working with TOPS therapists, either face to face or *via* telehealth. Sites without existing infrastructure for these services had to establish procedures and identify personnel who could perform those tasks.

Awareness of the program was a key issue identified across sites, and this was a particular issue when there was not an established follow-up clinic for these patients, but rather follow-up across various subspeciality clinics without embedded TOPS-trained providers. Potential referral sources also had difficulty recognizing who was eligible and determining who was appropriate to refer, particularly if there were significant comorbid mental health concerns. In the latter situation, anecdotal feedback indicated that they were more likely to use traditional mental health referrals, rather than refer to a novel program with which their institution lacked experience.

Given these Institutional barriers, the following strategies were identified:

• Establish strategies for routinely screening clinic schedules and flagging potentially eligible patients at sites with existing follow-up clinics, including use of EMR search features.
• Promote outreach (including TOPS training) for behavioral health providers embedded in these clinics so that they could support enrollment.
• Identify a coordinator who could screen clinic schedules and flag potentially eligible patients based on age and diagnoses supported this process, although this took time to implement and was not possible at all sites.
• Identify site-specific procedures for streamlining the process of delivering the program and minimizing additional administrative burdens.
• Engage leadership to support broader marketing initiatives to reach out to patients who may not be receiving follow-up care.

#### 3.6.2. Provider barriers

Although substantial numbers of potential providers received the TOPS training at each site, most did not plan to deliver the program because it was not part of their current workflow or role at the site (e.g., functioned in supervisory rather than direct care capacity). Since most of the patients with TBI/ABI were not currently receiving behavioral health services, offering care to them would require additional providers, which was not planned at most sites. COVID-19, and the mental health challenges arising from it, further exacerbated therapist shortages due to increased demand for services and fewer providers due to illness, resignation, or furlough.

Lack of provider buy-in or reluctance to refer patients to something new was also noted. This reluctance was characterized by one provider as follows: “To use a shopping analogy, our goal is to get great bread. We have multiple options we know and trust in our region. A newer brand may come on the market, and it might be tasty indeed, but if the barriers to getting it are basically the same, why switch?” Additionally, some providers expressed reluctance to refer patients to a self-guided program, and wanted to limit referrals to the therapist-involved options. Associated factors included perceptions that the program duplicated existing services or that the program wouldn't work in their setting because it was developed elsewhere. Remembering how to refer appropriate patients and finding time during a relatively brief outpatient visit to describe the program and make the referral constituted additional barriers. Specifically, although families expressed greater interest in the program when the provider had the opportunity to log on and demonstrate it to them, this was often precluded by tightly scheduled visits. Finally, providers often lacked the ability to follow-up and determine whether the family engaged with the program and to understand reasons for nonengagement.

Given these Provider barriers, the following strategies were identified:

• Develop brief presentations and informative fliers that site champions shared with brain injury and other subspecialty clinics, as well as other providers who were likely to come into contact with eligible patients.
• Tailor treatment delivery modes to further address barriers to care such as insurance coverage, therapist availability, and out-of-state residence.

#### 3.6.3. Patient/family barriers

Reasons families cited for declining treatment included the adolescent not wanting to identify as having issues related to their TBI/brain condition or the desire to prioritize another area (e.g., schoolwork, participating in sport). In some cases, these reasons may reflect the ongoing stigma associated with mental health treatment. For other families, general stressors (e.g., parent with cancer) took precedence over the brain condition or the child was already receiving treatment from community providers, and the family was reluctant to start an additional program, even if it was self-directed. The family focus of the treatment also led some families to decline due to high levels of family conflict, lack of parental time, or the desire to focus the treatment on the child. Some families expressed discomfort with the technical aspects of navigating the website or lacked broadband access; whereas others preferred a treatment that did not involve screens or that was more customized to their needs. Although traditional, face-to-face treatment was an option at all sites once pandemic restrictions relaxed, distance and available therapists continued to pose barriers. Families who wanted to work with a therapist were often deterred by therapist unavailability and long wait times for a first appointment. Limitations of insurance coverage, and out-of-state residence posed further barriers to obtaining either of the therapist options. For patients with a specific concern (e.g., anger management), there was reluctance to engage in a more comprehensive program that addressed issues in addition to their concern. Finally, although there was a Spanish language version of the TOPS website, adolescents with Spanish-speaking parents preferred the English version and primary languages other than English or Spanish were not supported.

Approximately one quarter of families who expressed interest in the program failed to follow through by creating a username and password, or received a username and password, but never accessed the program. Barriers identified by referral sources included lost log in information and difficulties updating their password, fatigue with screens/videoconferencing, and being overwhelmed by multiple stressors.

Given these Patient/Family barriers, the following strategies were identified:

• Increase patient awareness and linkages to the program through presentations to stakeholder organizations, such as brain injury and brain tumor support groups. Some sites worked to more closely integrate TOPS delivery into existing programs, making it a step-down program following initial outpatient rehabilitation.
• Link TOPS referrals to neuropsychological evaluations and feedback sessions to establish TOPS as a complement, rather than competitor, to existing services through increased education and collaboration with providers across subspecialties.
• Establish procedures for directly enrolling families during the child's clinic visit, thereby eliminating the gap between agreeing to enroll and creating a login/password. When time permitted, enrolling providers also previewed the sessions and showed parents and adolescents some of the skills they would learn.
• Tailor program delivery to address patient concerns. In practice, this meant shifting the order of session content to maximize relevance. For example, therapists could address emotion regulation or communication before working on executive functioning skills, for example, if that corresponded to patient and family concerns.

## 4. Discussion

Our implementation of TOPS led to the development of solutions to overcome challenges at the institutional, provider, and patient/family level; such solutions may be relevant to others seeking to implement EBPs for patients with low-incidence conditions and for those interested in providing a complex eHealth treatment program into clinical practice. To our knowledge, this is the first pediatric rehabilitation intervention to be broadly implemented, although efforts have been made to implement other evidence-based pediatric interventions such as Bright IDEAS (22). Our choice to implement through large children's hospitals allowed us reach children with a range of complex neurological conditions who might not otherwise receive evidence-based follow-up care. However, it also introduced complex institutional barriers that were further exacerbated by the COVID pandemic. Follow-up medical care for TBI and other complex neurological conditions can be siloed and focused on presenting concerns, making it difficult to connect patients who could benefit from the program. For example, headaches may be addressed by neurologists, while school issues are evaluated by neuropsychologists. An imbalance between therapist availability and clinical demand limited many sites' ability to offer the therapist-guided option. Patient and family barriers, including ambivalence about receiving behavioral health treatment for their concerns, have posed additional challenges. Anecdotally, the self-guided program may afford youth and families who are not ready to work with a psychologist an opportunity to begin to understand and address some of their concerns. Our collaborative model involving monthly cross-site meetings has been integral in identifying and exploring tailored solutions. Additionally, these meetings created an informal, national network of pediatric brain injury providers, allowing a broader sharing of experiences and resources. Given heterogeneity among our sites, solutions that are vetted at one site can be extended to other sites with similar clinical structures. We offer our experiences to inform future implementation efforts in the field of pediatric rehabilitation.

The COVID pandemic, which coincided with our implementation efforts, has offered unprecedented barriers and unique opportunities. Outpatient clinics were cancelled for a period of time and at some sites, the individuals who were trained to deliver the program were temporarily furloughed due to low patient volume. Institutions, providers, and patients faced demands and financial strains that made the introduction of a new program challenging and often unwelcome. However, the expansion of telehealth and Medicaid waivers to support provision of telehealth across state boundaries in some regions served as a catalyst for the provision of telehealth services at institutions that were previously resistant to such initiatives. Patient and family enthusiasm and engagement with telehealth waxed and waned over the course of the first two years of the pandemic. Although families initially embraced telehealth visits, therapists reported greater family fatigue with videoconferencing over time, particularly when both school and outpatient medical visits/therapies were virtual. As a consequence, some patients embraced the opportunity to resume in-person therapy sessions when restrictions were relaxed. Hybrid delivery models that included some virtual and some in-person visits also improved uptake. As challenges arising from the pandemic continued to evolve, we sought to adapt our approach to address these challenges. For example, we retrained therapists who were trained early in the pandemic and were subsequently furloughed before they could deliver the program and continued to explore program tailoring options to improve patient engagement (e.g., automatic reminders to complete online modules). Ultimately, it is not possible to fully disentangle the positive (e.g., great telehealth access) and negative impacts (e.g., reduced therapist availability, patient disengagement) of the COVID pandemic or the ultimate effectiveness of our responses.

During our initial two years of implementation, our reach was substantially lower than the two patients per site per month with TBI that was initially anticipated. Although COVID resulted in a number of unanticipated challenges and delays in ramping up, implementation within large, complex medical systems required considerable time to establish pathways and procedures before successfully enrolling patients. Inclusion of other neurological conditions with similar neurobehavioral consequences allowed us to substantially expand our reach, nearly doubling the numbers enrolled, and extend evidence-based practices to historically under-served patient populations. Consistent with other multisite implementations there continues to be considerable variability across sites suggesting the possibility to further extend reach as we more broadly implement the outreach strategies that we have identified (23).

Efforts to expand therapist adoption of the program (training and program delivery) have been more successful. As sites have established procedures for screening patients for appropriateness and referring them to the program, newly trained therapists can be more readily connected with patients on the waiting list. Thus, we are less likely to lose potential therapists to moves/completion of training before they have an opportunity to deliver the program or lose patients due to a lack of available TOPS-trained therapists. Positive preliminary feedback from SLPs who have successfully delivered the TOPS program suggests that this may be another avenue for extending program adoption. At another level, the first cohort of fellows who were trained in TOPS have recently begun jobs, potentially supporting further program spread. Although promising, it will take a few years to determine whether those who delivered the program during fellowship continue to use it in their new roles.

Implementation through large children's hospitals that are part of broader academic networks has afforded unique opportunities to extend the program to adolescents with a range of low-incidence conditions. However, it also posed challenges due to the complex and varied systems for tracking and managing children with ABI and other neurological conditions and the multiple subspecialties involved in their care. Heterogeneity across sites necessitated tailoring solutions to the institution, rather than relying on a one-size-fits-all approach. Thus, although successful solutions could be shared through our monthly All Teach All Learn format, adaptation to the local context was frequently required. Specifically, larger hospital systems often had more complex procedures for implementing new programs that required vetting and legal approval at a variety of levels; whereas, smaller systems were more nimble and responsive. Moreover, the pandemic exacerbated institutional challenges arising from understaffing and stretched resources.

At the patient level, we identified program-factors, such as lost logins/passwords and lack of reminders, that can be addressed to support engagement. However, factors such as negative attitudes toward behavioral health treatments or overall fatigue and burnout have proved harder to address. Additionally, complex comorbidities have raised issues among both parents and referral sources regarding how to sequence treatments and where TOPS fits in that sequence.

Our experiences to date should be considered in the context of a number of important limitations. As with any multisite implementation, there was inconsistent fidelity to critical elements and cross-site variability in some procedures. This included variable patient screening procedures, which made it difficult to accurately evaluate patient uptake and reasons for nonparticipation. Tracking of implementation metrics across sites has been variable based on site resources (e.g., a coordinator who can help collect and enter these data). Additionally, as with any multisite quality improvement project, sites varied in the exact timing of introduction of different improvement strategies across sites (e.g., one site trialed something and then when successful others followed). This made it challenging to evaluate the impact of each specific intervention on reach.

In summary, the initial two years of TOPS implementation have illustrated the complexity and challenges of moving an EBP of complex eHealth program into clinical care. They have also underscored the importance of a team-based approach and ongoing trouble-shooting/problem-solving. Future directions include creation of a sustainable online platform for training and extending the program to additional sites. Additionally, development of subsequent pediatric rehabilitation interventions could benefit from thinking about clinical implementation during the intervention development phase, thereby promoting longer-term institutional buy-in.

## Data Availability

The raw data supporting the conclusions of this article will be made available by the authors, without undue reservation.
